# Investigating irregularly patterned deep brain stimulation signal design using biophysical models

**DOI:** 10.3389/fncom.2015.00078

**Published:** 2015-06-26

**Authors:** Samantha R. Summerson, Behnaam Aazhang, Caleb Kemere

**Affiliations:** ^1^Department of Electrical Engineering and Computer Science, University of California, BerkeleyBerkeley, CA, USA; ^2^Department of Electrical and Computer Engineering, Rice UniversityHouston, TX, USA; ^3^Department of Neuroscience, Baylor College of MedicineHouston, TX, USA

**Keywords:** deep brain stimulation, Parkinson's disease, antidromic, computational modeling, firing rate entropy

## Abstract

Parkinson's disease (PD) is a neurodegenerative disorder which follows from cell loss of dopaminergic neurons in the *substantia nigra pars compacta* (SNc), a nucleus in the basal ganglia (BG). Deep brain stimulation (DBS) is an electrical therapy that modulates the pathological activity to treat the motor symptoms of PD. Although this therapy is currently used in clinical practice, the sufficient conditions for therapeutic efficacy are unknown. In this work we develop a model of critical motor circuit structures in the brain using biophysical cell models as the base components and then evaluate performance of different DBS signals in this model to perform comparative studies of their efficacy. Biological models are an important tool for gaining insights into neural function and, in this case, serve as effective tools for investigating innovative new DBS paradigms. Experiments were performed using the hemi-parkinsonian rodent model to test the same set of signals, verifying the obedience of the model to physiological trends. We show that antidromic spiking from DBS of the subthalamic nucleus (STN) has a significant impact on cortical neural activity, which is frequency dependent and additionally modulated by the regularity of the stimulus pulse train used. Irregular spacing between stimulus pulses, where the amount of variability added is bounded, is shown to increase diversification of response of basal ganglia neurons and reduce entropic noise in cortical neurons, which may be fundamentally important to restoration of information flow in the motor circuit.

## Introduction

Parkinson's disease (PD) is a neurodegenerative disorder which stems from dysfunction in the basal ganglia (BG), a group of deep brain nuclei that play a prominent role in the regulation of motor movement. This disorder follows the loss of dopaminergic neurons in the *substantia nigra pars compacta* (SNc), which projects to the striatum, and the subsequent disrupted balance of excitatory and inhibitory activity in the downstream structures. Although deep brain stimulation (DBS) may be used to treat motor symptoms of PD, there remains a poor mechanistic understanding of the therapeutic action of electrical modulation of the neural activity in the BG and other nuclei in the motor circuit.

Increased coherence in BG structures and power in the beta frequency band (13–30 Hz) are prominent biomarkers of parkinsonian activity. Dopamine-dependent beta band coherence between the cerebral cortex and the *subthalamic nucleus* (STN) has been found in humans with PD (Williams et al., 2002) and in the parkinsonian rodent model (Sharott et al., 2005; Mallet et al., 2008a). Amplified activity in the beta band has been found in various BG nuclei, including the STN and *globus pallis interna* (GPi), as well as in the striatum and motor cortex (Bergman et al., 1994; Brown et al., 2001; Goldberg et al., 2002; Kuhn et al., 2005; Williams et al., 2005; Mallet et al., 2008b).

Excessive synchronization also occurs at the level of single neuron activity and significant changes have been observed in the firing properties of single cells. In the MPTP non-human primate model of PD it has been shown that firing rates of GPi and STN neurons increase, while the firing rates of neurons in the *globus pallidus externa* (GPe), thalamus, and motor cortex decrease (Miller and DeLong, 1988; Filion and Tremblay, 1991; Bergman et al., 1994; Schneider and Rothblat, 1996; Elder and Vitek, 2001; Pasquereau and Turner, 2011). Additionally, discharges tend to occur more frequently in bursts (Miller and DeLong, 1988; Filion and Tremblay, 1991; Bergman et al., 1994; Hutchinson et al., 1994; Magnin et al., 2000).

Much progress has been made toward understanding what changes to firing patterns, coherence and other neurological attributes are induced by the stimulation, but the sufficient conditions for therapeutic efficacy are unknown and the optimal stimulation strategy remains an open question. In rodents it was shown that high frequency stimulation (HFS) of the STN reduced low-frequency coherence within and across the GPe and *substantia nigra pars reticulata* (SNr; McConnell et al., 2012). Additionally high frequency DBS of the STN reduces beta band spike-field coherence in M1 (Li et al., 2012). Human studies have found that DBS attenuates beta band power in deep brain and cortical structures (Kuhn et al., 2008; Whitmer et al., 2012). These results that have been confirmed in animal models as well, where increasing stimulation frequency has been shown to be correlated with greater attenuation of beta power (Li et al., 2012; McConnell et al., 2012). Stimulating the STN of MPTP non-human primates has been shown to induce specific changes in the temporal firing patterns of neurons, namely the pattern of discharge became more regular, in the GPe and GPi which have been linked to the therapeutic efficacy (Hashimoto et al., 2003; Moran et al., 2011).

Previous theories about the mechanism of action of DBS centered on the idea of regularizing pathological activity through entrainment and synaptic modifications (Rubin and Terman, 2004; Perlmutter and Mink, 2006; Birdno et al., 2007; Dorval et al., 2010). However, further studies have revealed that DBS of the STN causes mixture of changes in firing rate in efferent structures (Hashimoto et al., 2003; Shi et al., 2006; Reese et al., 2011; Humphries and Gurney, 2012). Computational studies have elucidated frequency-dependent effects on diversification of the firing rates in these basal ganglia structures (Humphries and Gurney, 2012; Summerson et al., 2014a). In addition to the effect that orthodromic activity has on therapeutic efficacy, antidromic activity on the hyperdirect pathway between the motor cortex and STN has also been shown to impact treatment potency (Li et al., 2007, 2012; Kang and Lowery, 2014). In order to address how frequency and regularity of the stimulation pattern impact both antidromic and orthodromic activity, we develop a model of critical motor circuit components in the brain using biophysical cell models as the basic units and then evaluate performance of different DBS signals in this model to perform comparative studies of their efficacy. We hypothesize that allowing the stimulus pulses occur at irregular intervals, as long as there are never long windows without stimulation, may be more effective in reducing pathologically coherent activity and in diversifying neural responses to stimulation.

We develop system-level models of important BG structures, as well as the output layer (layer V) of the primary motor cortex (M1) and the thalamus, which relays information from the BG to the cortex. These models are used to examine new stimulation signal designs and their influence on firing rate changes and patterns. Biological models are an important tool for gaining insights into neural function and, in this case, serve as an effective test bed for innovative DBS paradigms. The integrity of the model is confirmed through comparison with *in vivo* recordings acquired from hemi-parkinsonian rats receiving DBS through chronically implanted microelectrodes.

## Materials and methods

Biophysical models, along the lines of conductance-based Hodkin-Huxley cell models, provide a mathematical description of how synaptic, ionic, and injected currents influence the membrane voltage potential over time through a set of differential equations. Single-compartment cell models with multiple input currents are used here to model the spiking behavior of neurons which serves as the basic components in a larger scale model of structures within the motor circuit of the brain. By adjusting parameters we can model properties exhibited in a variety of phenotypic states, namely neural activity in the healthy, parkinsonian and parkinsonian with DBS states. Six cell types are modeled here: cortical pyramidal cells, cortical interneurons, STN cells, GPe, and GPi cells, and thalamic neurons. The intrinsic ionic current due to ion *j* flow across the membrane of cell *i* is represented by the form:

$$I_{j,i}\;=\;\bar{g_{j}}m_{j}^{M}\;h_{j}^{N}\;(V_{i}-E_{j})$$

where the variable *g_j_* is the maximal conductance for ion *j*, and *m_j_* and *h_j_* are the activation and inactivation variables, respectively. In the following, the above current may be notated as *I_j_*, dropping subscript *i* when the cell type is clear. The single cell models are based on previous works, though parameter adjustments have been made to accommodate the network architecture described in the following section and conform to the spiking activity of experimental data. Additionally, we add small noise currents to the individual cells to account for inputs that are not explicitly modeled.

### Cortical model neurons

**Figure 1:**
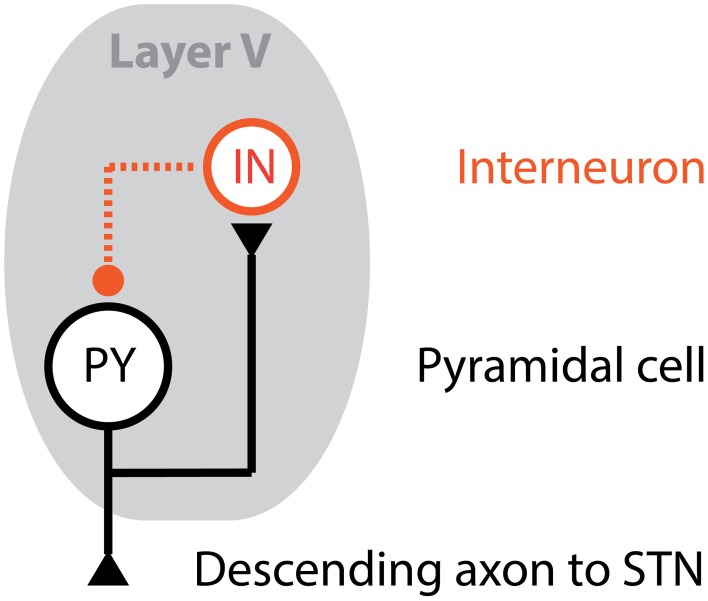
**Cortical unit computational model**. The output layer, Layer V, of the motor cortex is modeled using a population of cortical units, where each cortical unit is formed using a pyramidal cell (PY) and interneuron (IN) in a feedback architecture. The axon of the PY cell projects to the STN, while an axonal branch projects to the IN cell. In turn, the IN cell synapses onto the PY cell providing an inhibitory input.

The two main cell types that make up the cortex are excitatory pyramidal cells (PY) and inhibitory interneurons (IN). Both cell types are modeled here using single compartment models based on prior work (Destexhe et al., 1998), but with parameters tuned to match *in vivo* data. A recurrent network architecture, shown in Figure 1, is formed using a PY and IN model to replicate physiological findings. Stimulation of STN neurons excites both cell types: the PY cell body directly and the IN cell synaptically via the PY axonal branch.

The membrane potential of a PY cell, *V_PY_*, is described by:

$$\begin{array}{l} C_{m}\frac{dV_{PY}}{dt}=-g_{L}\left(V_{PY}-E_{L}\right)-I_{Na}-I_{K}-I_{M}\\ \;-\;I_{IN\to PY}-\;I_{DBS}-I_{z}, \end{array}$$

where *C_m_* is the membrane capacitance, *g_L_* is the leak conductance, *E_L_* is the leak voltage, *I_Na_* is the sodium current, *I_K_* is the potassium current, and *I_M_* is a slow voltage-dependent potassium current. For all PY cells, the maximal conductance values for the leak, sodium, potassium, and slow voltage-dependent potassium currents are 0.1, 50, 5, and 0.07 mS/cm^2^, respectively. The synaptic current *I_IN→PY_* is the input from the concomitant IN cell, the current from stimulation is denoted as *I_DBS_* and the small noise current is *I_Z_*.

The membrane potential of an IN cell, *V_IN_*, is described by:

$$C_{m}\frac{dV_{IN}}{dt}=\;-g_{L}\left(V_{IN}-E_{L}\right)-I_{Na}-I_{K}-I_{PY\to IN}-I_{Z},$$

where the notation used is consistent with previous definitions. The maximal conductance values for the leak, sodium, and potassium currents are 0.15, 50, and 5 mS/cm^2^, respectively. The synaptic input along the PY cell axonal branch is *I_PY→IN_*, which is a function of orthodromic activity from the PY cell as well as the DBS current pulses propagating antidromically if present.

### STN model neuron

The voltage across the membrane of a STN model neuron is determined by various ionic currents, as well as from the inhibitory synaptic input of GPe cells and DBS current when present. For the STN neurons, a notion of distance is also introduced into the model. It has been shown that the voltage potential change induced by a current pulse decays as a function of the distance between the neuron and the current source (Rattay, 1999; Miocinovic et al., 2006). Thus, we uniformly distributed the STN neurons inside a sphere of radius *r*, where the DBS electrode is defined to be located at the center of the sphere. Since the STN can be approximated as an ellipsoid with the smallest axis of length around 4 mm (Richter et al., 2004), we assume that *r* = 4 mm for the sphere. If a neuron is located d mm from the current source, the amplitude of the current is attenuated by an exponential function of the distance so that the current seen at the neuron is

$$I_{DBS}=I_{stim}e^{-d}.$$

The voltage equation for the membrane potential, *V_STN_*, of a STN model neuron is based on previous work (Rubin and Terman, 2004) and defined by:

$$\begin{array}{l} C_{m}\frac{dV_{STN}}{dt}=-g_{L}\left(V_{STN}-E_{L}\right)-I_{Na}-I_{K}-I_{T}-\;I_{Ca}\\ \;-\;I_{GPE\to STN}-I_{DBS}-I_{Z}, \end{array}$$

where *I_T_* is a T-type low-threshold spiking current, *I_Ca_* is a calcium spiking current and *I*_GPE→STN_ is the cumulative inhibitory synaptic current from afferent GPe model neurons. The maximal conductance values for the leak, sodium, potassium, low-threshold calcium and calcium channels are 2.25, 37.5, 45, 0.5, and 0.15 mS/cm^2^, respectively.

### GPe and GPi model neurons

The model neurons for the GPe and GPi are very similar. The intrinsic ionic currents are the same for both model types, though their afferent connections differ causing discrepancies in the synaptic currents that influence the membrane voltage. These two models are also based on previous work (Rubin and Terman, 2004). The voltage equation for the membrane potential, *V_GPe_*, of a GPe model neuron is:

$$\begin{array}{l} C_{m}\frac{dV_{GPe}}{dt}=-g_{L}\left(V_{GPe}-E_{L}\right)-I_{Na}-I_{K}-I_{T}-I_{Ca}\\ \;-\;I_{STN\to GPe}-I_{GPe\to GPe}-I_{str}-I_{Z,} \end{array}$$

where the maximal conductance values for the sodium, potassium, low-threshold calcium and calcium currents are 0.1, 120, 30, 0.5, and 0.15 mS/cm^2^, respectively. The excitatory input from afferent STN cells is denoted as *I*_*STN*→*GPe*_ and inhibitory input from other GPe model neurons is denoted as *I*_*GPe*→*GPe*_. The input to the GPe neurons from the striatum is modeled as a constant current, *I_str_*.

The GPi neurons are modeled similarly, with the voltage equation for the membrane potential, *V_GPi_*, of a GPi model neuron represented by:

$$\begin{array}{l} C_{m}\frac{dV_{GPi}}{dt}=-g_{L}\left(V_{GPi}-E_{L}\right)-I_{Na}-I_{K}-I_{T}-I_{Ca}\\ \;-\;I_{STN\to GPi}-I_{app}-I_{Z}, \end{array}$$

where the maximal conductance values for the ionic channels are the same as for the GPe model neurons. The GPi cells also receive excitatory input from the STN, but there are no recurrent connections within the nucleus. Additionally, a constant current, *I_app_*, is applied in order to ensure that the intrinsic firing rate of the GPi neurons is higher than GPe neurons to be consistent with experimental data (Hashimoto et al., 2003).

### Thalamocortical model neuron

The final cell type included in our large scale model is for thalamocortical (TC) cells. The main output of the basal ganglia is the GPi, projecting to the thalamus which in turn relays signals to the cortex. The voltage equation for the membrane potential, *V_TC_*, of a TC cell is

$$\frac{dV_{TC}}{dt}=\;-g_{L}\left(V_{TC}-E_{L}\right)-I_{Na}\text{​}-\text{​}I_{K}-I_{T}-I_{e}-I_{GPi\to TC}-I_{Z},$$

where the maximal conductance values for the leak, sodium, potassium, and low-threshold calcium channels are 0.05, 3, 5, and 5 mS/cm^2^, respectively. The current *I_e_* represents time-varying excitatory synaptic inputs from cells not explicitly represented. TC cells also receive inhibitory input from GPi neuron which hyperpolarizes the cell membrane.

### Network model and synaptic connectivity

Each nucleus is represented by population of 16 model neurons and intra- and inter-nuclei connections are defined to build a model of the entire cortico-basal ganglia circuit. As previously mentioned, the PY cells project to a single cortical IN cell and STN cell, with the IN cell connected in a feedback architecture. The remaining synaptic connections for basal ganglia and TC cells are randomly generated at the beginning of the simulation, with the strength of these connections evolving over time according to the synaptic conductivity differential equations. The connections are initiated by assuming each cell *y* receives a fixed number *n_x,y_* of inputs from a presynaptic cell type *x*. This is done independently for each cell *y*. We chose the number of inputs per cell type, *n_x,y_*, to match empirical data and previous reports of approximate connectivity density between nuclei (Rubin and Terman, 2004; Humphries and Gurney, 2012), and the values are presented in Table 1.

**Table 1:** **Synaptic connections between cells**.

**Presynaptic cell (x)**	**Postsynaptic cell (y)**	**Number of connections (*n_x,y_*)**
GPe	STN	2
GPe	GPe	2
GPe	GPi	2
STN	GPe	3
STN	GPi	1
GPi	TC	2

*Synaptic connections are randomly generated between model presynaptic and postsynaptic cells, with the number of incoming connections to a postsynaptic cell indicated in the right column*.

**Figure 2:**
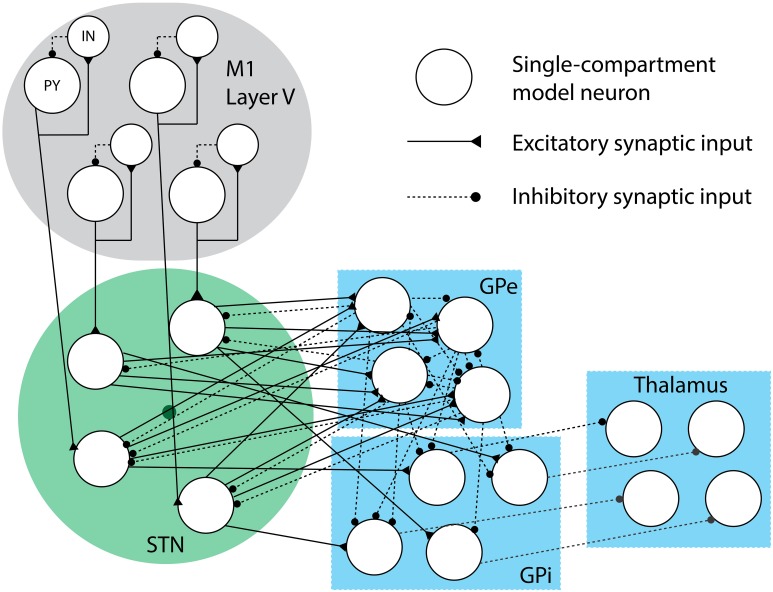
**Synaptic connectivity cartoon**. This small-scale network of model neurons depicts the formation of each model nucleus and the synaptic connections. Excitatory synaptic connections are represented with sold lines, while inhibitory synaptic connections are represented with dashed lines. The dark circle in the middle of the STN neurons represents the DBS electrode, which the neurons are randomly distributed around in a sphere of radius *r* (drawn in green).

The connectivity assignments were selected according to a uniform distribution between it provides maximal randomness in the model of connectivity, which is most appropriate when using a small number of model neurons to approximate activity from a structure with orders of magnitude more cells. The *n_x,y_* indices are selected by sampling uniformly without replacement from the index set of all presynaptic cells of type *x*. Since this is done independently for each postsynaptic cell, the postsynaptic cells are permitted to have common inputs. To illustrate the connectivity model assumed, a small example network with four cells per nucleus is depicted in Figure 2.

### Stimulation patterns

To simulate the effect of DBS on the model network, the STN neurons receive a current input that represents injected current from a microelectrode located at the center of the STN model neuron modulation. In practice, current pulses are biphasic, so current flow alternates directions between two electrical contacts to ensure that charge does not accumulative in neural tissue and cause irreversible damage. The DBS electrode here is modeled as a point source with the current signal consisting of a train of current pulses of width ω seconds and amplitude αpA/μm^2^. One pulse, *p*_α_ (*t*), can be described as:

$$p_{\alpha }(t)=\;\{\begin{array}{cc} -\alpha & \text{0}\leq t<\omega \\ 0 & otherwise \end{array}.$$

Hence, an infinite train of such pulses can represented as:

$$I_{stim}(t)=\sum_{n\;=\;0}^{\infty }p_{\alpha }\left(t-\frac{n}{f}\right),$$

where *f* is the stimulation frequency. To create an irregular pattern of stimulation, bounded noise is added to the timing of these pulses. Irregular DBS current, *I^irr^_stim_*(*t*), can be written as:

$$I_{stim}^{irr}(t)=\sum_{n\;=\;0}^{\infty }p_{\alpha }\left(t-z_{n}-\frac{n}{f}\right),$$

where *z_n_* are i.i.d. uniform random variables over the range [−*s, s*] for some *s* ϵ ℝ for all *n*. The period is irregular and stochastic, but the average inter-pulse period between pulses is still *1/f* and the maximum and minimum time between pulses is bounded, ensuring that the average rate of pulses is high and there are never long periods between pulses.

### *In vivo* experiments

The 6-hydroxydopamine (6-OHDA) rodent model has frequently been used to study PD and DBS (Sharott et al., 2005; Li et al., 2012; McConnell et al., 2012; Summerson et al., 2014b) where injections are typically given unilaterally to create a hemi-Parkinsonian rodent model. Previous work has demonstrated the influence of regular DBS of the STN on firing properties of BG nuclei (McConnell et al., 2012) and the primary motor cortex (M1) via the hyperdirect pathway (Li et al., 2012), which was the first report of M1 activity in awake, freely moving hemi-Parkinsonian rats receiving DBS. In a population of 6-OHDA rats (*n* = 6), we stimulated the STN using regular and irregular stimulation patterns at two frequencies in order to capture frequency- and regularity-dependent effects of stimulation. Subjects behaved spontaneously and both single-unit and local field potential (LFP) activity was captured bilaterally during the DBS sessions.

The amplitude and pulse width of the current pulses were 100 μA and 60 μs, respectively, which are parameters that have previously been established as effective (McConnell et al., 2012; Summerson et al., 2014b). Two different stimulation frequencies were tested: 40 and 130 Hz. This set of frequencies consists of an untherapeutic therapeutic value (40 Hz) and a highly therapeutic value (130 Hz) values. For all *n*, *z_n_* was a uniform random variable over the interval [−1, 1] ms, with a resolution of 2 μs resolution dictated by the technical specifications of the stimulator. The LFP signal was captured at a sampling rate of 30 kHz. Individual units were identified from threshold-crossing events and recorded as an array of 40 data samples collected at a rate of 10 kHz from the digitally filtered raw signal. Offline analysis was perform to remove the stimulation artifact from the LFP signal and additional filtering for examining power spectrum measurements. All experiments were approved by the Institutional Animal Care and Use Committee (IACUC) of Rice University.

## Results

Activity from the model structures was simulated under various conditions to ascertain changes induced from stimulation frequencies using stimulation patterns that were both regular and irregular. Simulated M1 activity is compared to neural activity acquired from a population of hemi-parkinsonian rats receiving unilateral STN DBS.

### Firing rate changes for BG cells

**Figure 3:**
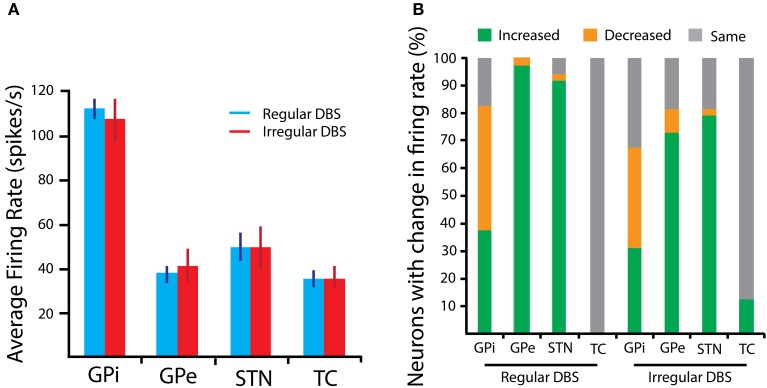
**BG Firing rate properties in response to 130 Hz DBS. (A)** The average firing rate, computed across the population of model neurons representing a nucleus, is shown for each structure. Error bars are SEM. **(B)** The percentage of neurons with either an increased, decreased, or unchanged firing rate relative to the spiking properties in the parkinsonian state is presented. Changes are more heterogeneous in response to the irregular stimulus pulse train.

The firing rate of all cells in the modeled BG structures was computed and averaged across each structure to characterize the average change of single-unit activity. For each nucleus in the basal ganglia and for the TC cells, there was no significant difference in average firing rate when 130 Hz was applied to the model with regular and irregular pulse trains (One-Way MANOVA: *p* > 0.05; see Figure 3A). Even though the average rate across the nuclei did not differ when random perturbations were added to the pulse times, it was found that DBS has a non-uniform effect on spiking properties of individual neurons. This is discussed further in the next section.

### Mixture of response

The mixture of response to the stimulation, namely the proportion of neurons with increased, decreased and unaffected firing rates, has been previously shown to be related to therapeutic efficacy of DBS (McCairn and Turner, 2009; Humphries and Gurney, 2012; McConnell et al., 2012). One hypothesis is that this mixture is essential to establish balance between regularization and inhibition of GPi neurons, which serve as the main output of the basal ganglia (Humphries and Gurney, 2012). Irregular stimulation produces greater variation in the number of neurons with up- and down-modulated firing rates, as shown in Figure 3B for 130 Hz stimulation. This is particularly true of GPi neurons, where inhibition counterbalancing regularization may be principally important to restoration of information flow out of the basal ganglia. This mixture in the change in firing rate of GPi neurons is a result of its afferent neurons, namely from STN and GPe, which also experience divergent responses as a result of orthodromic modulation from the stimulation.

### Antidromic spiking and entropic noise

**Figure 4:**
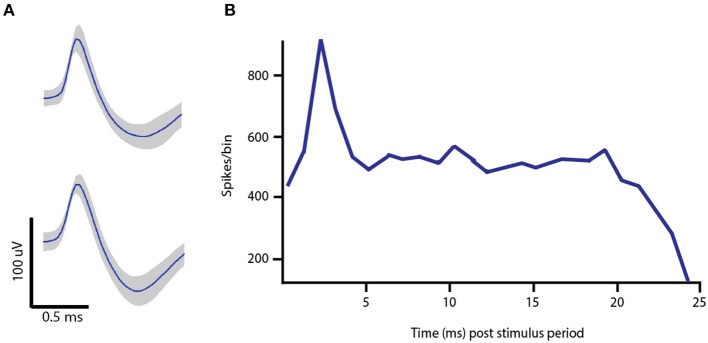
***In vivo* single-unit data. (A)** Example spike waveforms recorded from Layer V of M1 in the lesioned hemisphere of a 6-OHDA hemi-parkinsonian rat. **(B)** Peristimulus time histogram of pyramidal cell spiking activity in response to 130 Hz DBS with regularly space current pulses. The peak around 2–3 ms is attributed to the fact that antidromic spikes appear at the cell body with a short, fixed latency following the DBS pulse onset.

Descending axons from the output layer of the cortex to the STN form the so-called hyperdirect pathway. When administering DBS in the STN, the injected current can activate both afferent and efferent axons (Hashimoto et al., 2003; Hammond et al., 2008). Previous work has shown that antidromic and orthodromic modulation of cortical activity may be important for therapeutic benefit (Dejean et al., 2009; Gradinaru et al., 2009; Kang and Lowery, 2014), and that stimulation of STN indeed evokes antidromic spikes in the output layer of motor cortex (Li et al., 2012). Antidromic spikes originate in the axon of the efferent STN cells and propagate toward the body of the presynaptic cell, arriving with a short fixed latency from the time of a stimulus pulse. In Figure 4, example spike waves are presented, as well as the peristimulus time histogram (PSTH). The peak around 2–3 ms is due to the antidromic spiking activity.

A frequency-dependent relationship between the fidelity of antidromic spikes to stimulus pulse has been found (Li et al., 2012). With increasing stimulation frequency, there is a monotonic decrease in the fidelity of pyramidal projection neurons to the stimulus pulse. When taken in conjuction with the frequency of stimulation, the resulting behavior for the antidromic spike rate as a function of stimulation frequency has a concave form. Our model of the network of Layer V cells in M1 that experience modulatory effects from DBS reproduce this trend found in Li et al. and agrees with our own *in vivo* recordings for the two stimulation frequencies tested. In the model, the firing rate of INs increases monotonically with the stimulation frequency, which is expected since there are no additional inputs in the model to inhibit activity. While the excitatory antidromic input to a PY cell increases with frequency, so does the inhibitory synaptic input from an afferent IN cell. In order to reproduce *in vivo* behavior, the strength of the synaptic conductivity for the IN to PY synapse is the key parameter that is tuned to achieve the required level of inhibition at the PY cell. Results are more robust to variations in the synaptic conductivity from the PY to IN cell, the amplitude of the DBS current pulse arriving at the PY soma and IN synapse, though all of these variables were considered jointly when tuned to fit the data.

**Figure 5:**
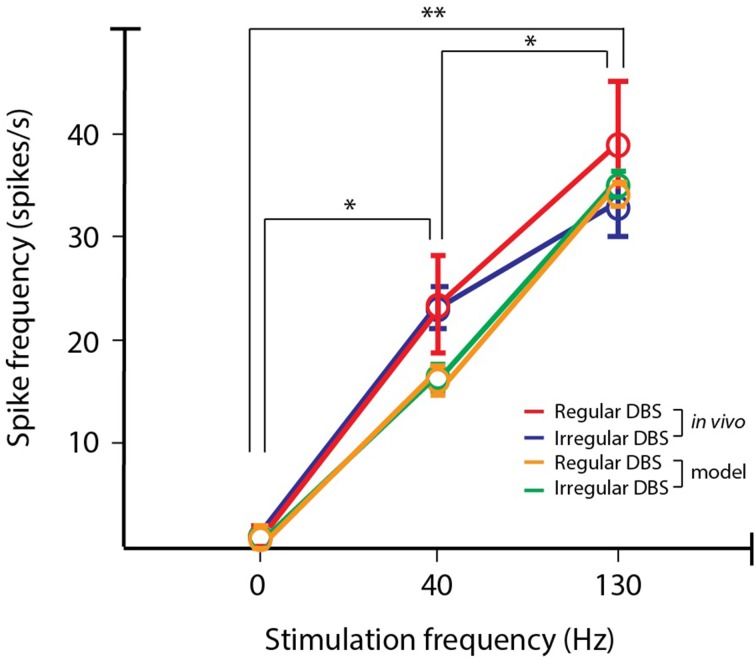
**PY firing rate for *in vivo* and simulated data**. The firing rate of cell is in the output layer of the motor cortex is antidromically modulated by STN DBS. The modeled PY cells reproduce the same trends of differential modulation as found from recordings in the hemi-parkinsonian rodent model. In both the observed data and model data, increasing stimulation frequency produced increases in firing rates (*post-hoc* LSD: ^*^p < 0.05, ^**^p < 0.01).

The resultant firing rate of the PY cells was computed for the computational model and *in vivo* recording data, and is shown in Figure 5. As the stimulation frequency increased, the firing rate also increased, and the computational model data faithfully represents this behavior. As a measure of model fidelity, we consider the Pearson correlation coefficient between the observed and modeled data. Spike rate with regular stimulation was significantly correlated between *in vivo* and computational model data (ρ = 0.99, *p* < 0.05), as well as for spike rate measured in response to irregular stimulation (ρ = 0.96, *p* < 0.05). For all stimulation frequencies, there was no significant difference in modulatory effect on average firing rate of the cells produced by regular vs. irregular stimulation patterns (One-Way ANOVA: *p* > 0.05). However, the spike patterns were differentially altered as a function of frequency and regularity of the stimulus pulse train. To characterize this, we computed the entropy of the interspike intervals (ISIs) for the orthodromic spikes from the PY cells. For a discrete random variable *x*, this entropy can be written as:

$$H(x)\;=\;-\sum_{i}p(x_{i})\;\log_{2}\;p(x_{i}).$$

**Figure 6:**
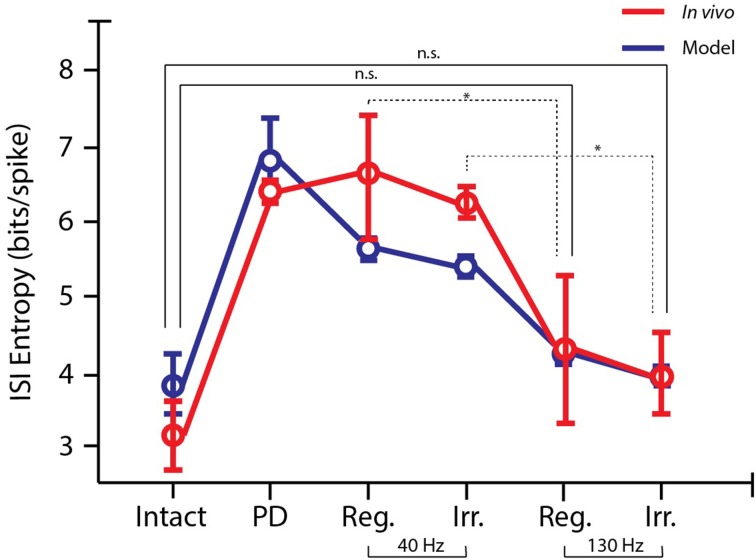
**Entropy of ISIs for *in vivo* and simulated data**. The activity produced by the computational model (*n* = 50, blue) follows the same trends as the *in vivo* data (*n* = 48, red). Spike trains are more bursty in the parkinsonian state, but the bursiness decreases with increasing stimulation frequency (*post-hoc* LSD: ^*^p < 0.05). 130 Hz regular and irregular DBS both restored the ISI entropy to the level of the intact hemisphere, meaning the ISI entropy was not significantly different (n.s.; *post-hoc* LSD: *p* > 0.05).

The entropy describes how much uncertainty or randomness there is in the variable *x*, which in these case represents the ISIs. If the firing is more regular, the entropy will be lower, whereas if the firing is more bursty, the entropy will be higher. The model data is correlated with the observed data (ρ = 0.91, *p* < 0.025), and in both cases there was a significant decrease in ISI entropy between 40 and 130 Hz stimulation (One-Way ANOVA: *p* < 0.01; *post-hoc* LSD: *p* < 0.05; see Figure 6). For each frequency, there was no significantly difference in the ISI entropy when random perturbations were added to the pulse train, meaning the pyramidal cell ISI entropy for both frequencies was maintained when random perturbations were added (*post-hoc* LSD: *p* > 0.05). Additionally, 130 Hz regular and irregular stimulation achieved ISI entropies that were not significantly different than the cell activity in the intact hemisphere (*post-hoc* LSD: *p* < 0.05).

### Beta-band power

**Figure 7:**
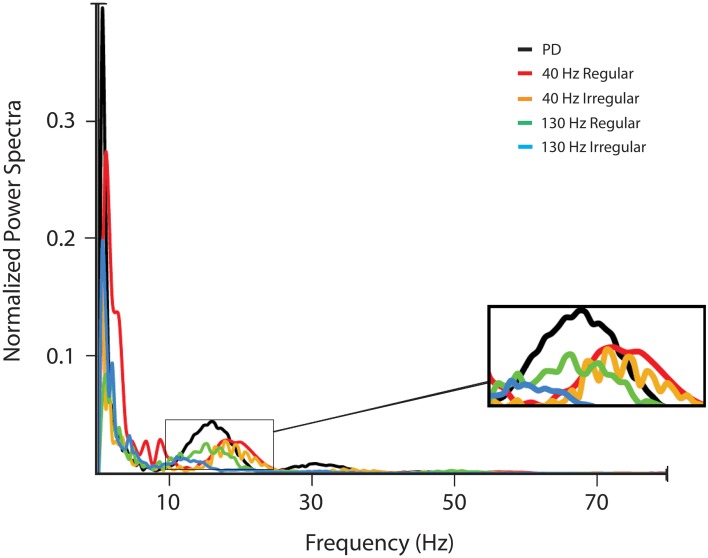
**LFP power spectra**. The LFP was filtered using a zero-phase FIR filter with passband 0–200 Hz and the power spectrum of the LFP signal was estimated using multitaper methods (Chronux 2.0) with 9 Slepian data tapers. Power spectra shown are for activity in the lesioned hemisphere of the subjects without stimulation and with four different stimulation signals: 40 Hz with regularly and irregularly space pulse trains, and 130 Hz DBS with regularly and irregularly spaced pulse trains.

The power spectrum of the observed LFP data during stimulation was computed in order to identify effects of the STN DBS in the rodent model on beta band activity, since increased beta band activity has been correlated with motor symptoms associated with PD (Brown et al., 2001). The averaged normalized spectrum is presented in Figure 7. With increasing stimulation frequency, beta band power was found to be increasingly attenuated (One-Way ANOVA: *p* < 0.05; *post-hoc* LSD: *p* < 0.025). Also, irregular stimulation dampened peak beta band power more than regular stimulation (*post-hoc* LSD: *p* < 0.05). These results confirm that pathological activity was reduced with high frequency, irregular stimulus pulse trains, as indicated by the computational model.

## Discussion

The large scale computational model enables investigation of the relationship between signal features, such as frequency and regularity of pulses, and changes in neuron firing properties. The standard DBS signal consists of a series of regularly spaced current or voltage pulses. It has widely been shown that high frequency regular stimulation (>100 Hz) is effective in treating motor symptoms of PD, whereas low frequency regular stimulation is not effective. Although therapeutic benefit is achieved using regularly spaced pulses, the neural activity is not restored to its non-pathological state and stimulated neurons tend to become pulse-locked, creating a narrowband increase in power in the frequency band around the stimulation frequency.

The temporal pattern of stimulus pulses strongly influences the performance of the treatment. Previous work has found that if the inter-pulse time periods are randomly distributed and the density governing the periods allows for long times between pulses, the stimulation is less effective (Dorval et al., 2010). We proposed generating a stimulation signal with irregularly spaced stimulus pulses by adding a random perturbation to each stimulus pulse time. The support of the random perturbation density is small, bounding the longest amount of time between pulses. In this way we ensure that even over small time windows the average stimulation frequency is high but the stimulated neurons no longer fire at fixed intervals.

Using the irregular DBS signal, we found a greater mixture of response in the change in firing rates of the BG neurons that were simulated when compared to using regular DBS of the same average stimulation frequency. This mixture of response, i.e., heterogeneity in the average increase or decrease of a neuron's firing rate, has previously been linked to efficacy of DBS treatment. Both regular and irregular stimulation antidromically modulated activity of the PY cell and reduced ISI entropy. In the Parkinsonian state, cortical cells are more bursty, which means the ISI entropy is high, and irregular DBS maintains the reduction in entropic noise achieved with regular DBS. These results faithfully reproduce *in vivo* activity characterized by the authors in hemi-parkinsonian rodents with DBS administered unilaterally. In the rodent model, the reduced ISI entropy also coincided with beta band power attenuation, which is associated with improvement in motor symptoms of PD. The construction of essential components in the motor circuit using the biophysical model neurons presented is validated as a potent tool for investigating Parkinsonian activity and promotes the investigation of further stimulation signal design principles for DBS therapy.

### Conflict of interest statement

The authors declare that the research was conducted in the absence of any commercial or financial relationships that could be construed as a potential conflict of interest.
