# Supplementary material for: Investigating irregularly patterned deep brain stimulation signal design using biophysical models
Source: Front Comput Neurosci. 2015 Jun 26;9:78. doi: 10.3389/fncom.2015.00078 (PMC4481153; doi:10.3389/fncom.2015.00078)
Supplement: Supplementary file 1 [file DataSheet1.DOCX]

1. **Appendix I: Model Parameter Values**

**Ionic current parameters**

The following values were used in the generation of the various model cell types that were simulated.

| Parameter | Value |
| --- | --- |
| ${\bar{\boldsymbol{g}}}_{\boldsymbol{L}}$ | 0.1 mS/cm^2^ |
| $\boldsymbol{E}_{\boldsymbol{L}}$ | -70 mV |
| ${\bar{\boldsymbol{g}}}_{\boldsymbol{Na}}$ | 50 mS/cm^2^ |
| ${\bar{\boldsymbol{g}}}_{\boldsymbol{K}}$ | 5 mS/cm^2^ |
| ${\bar{\boldsymbol{g}}}_{\boldsymbol{M}}$ | 0.07 mS/cm^2^ |
| $\boldsymbol{I}_{\boldsymbol{z}}$ | ~$N\left( 0,0.1 \right) uA$ |

**PY cells**

| ${\bar{\boldsymbol{g}}}_{\boldsymbol{L}}$ | 0.15 mS/cm^2^ |
| --- | --- |
| $\boldsymbol{E}_{\boldsymbol{L}}$ | -70 mV |
| ${\bar{\boldsymbol{g}}}_{\boldsymbol{Na}}$ | 50 mS/cm^2^ |
| ${\bar{\boldsymbol{g}}}_{\boldsymbol{K}}$ | 5 mS/cm^2^ |
| $\boldsymbol{I}_{\boldsymbol{z}}$ | ~$N\left( 0,0.1 \right) uA$ |

**IN cells**

| ${\bar{\boldsymbol{g}}}_{\boldsymbol{L}}$ | 0.1 mS/cm^2^ |
| --- | --- |
| $\boldsymbol{E}_{\boldsymbol{L}}$ | -55 mV |
| ${\bar{\boldsymbol{g}}}_{\boldsymbol{Na}}$ | 120 mS/cm^2^ |
| $\boldsymbol{E}_{\boldsymbol{Na}}$ | 55 mV |
| ${\bar{\boldsymbol{g}}}_{\boldsymbol{K}}$ | 30 mS/cm^2^ |
| $\boldsymbol{E}_{\boldsymbol{K}}$ | -80 mV |
| ${\bar{\boldsymbol{g}}}_{\boldsymbol{Ca}}$ | 0.15 mS/cm^2^ |
| $\boldsymbol{E}_{\boldsymbol{Ca}}$ | 120 mV |
| ${\bar{\boldsymbol{g}}}_{\boldsymbol{T}}$ | 0.5 mS/cm^2^ |
| ${\bar{\boldsymbol{g}}}_{\boldsymbol{AHP}}$ | 30 mS/cm^2^ |
| $\boldsymbol{I}_{\boldsymbol{str}}$ | -5 uA |
| $\boldsymbol{I}_{\boldsymbol{z}}$ | ~$N\left( 0,0.1 \right) uA$ |

**GPe cells**

| ${\bar{\boldsymbol{g}}}_{\boldsymbol{L}}$ | 0.1 mS/cm^2^ |
| --- | --- |
| $\boldsymbol{E}_{\boldsymbol{L}}$ | -55 mV |
| ${\bar{\boldsymbol{g}}}_{\boldsymbol{Na}}$ | 120 mS/cm^2^ |
| $\boldsymbol{E}_{\boldsymbol{Na}}$ | 55 mV |
| ${\bar{\boldsymbol{g}}}_{\boldsymbol{K}}$ | 30 mS/cm^2^ |
| $\boldsymbol{E}_{\boldsymbol{K}}$ | -80 mV |
| ${\bar{\boldsymbol{g}}}_{\boldsymbol{Ca}}$ | 0.15 mS/cm^2^ |
| $\boldsymbol{E}_{\boldsymbol{Ca}}$ | 120 mV |
| ${\bar{\boldsymbol{g}}}_{\boldsymbol{T}}$ | 0.5 mS/cm^2^ |
| ${\bar{\boldsymbol{g}}}_{\boldsymbol{AHP}}$ | 30 mS/cm^2^ |
| $\boldsymbol{I}_{\boldsymbol{app}}$ | 3 uA |
| $\boldsymbol{I}_{\boldsymbol{z}}$ | ~$N\left( 0,0.1 \right) uA$ |

**GPi cells**

| ${\bar{\boldsymbol{g}}}_{\boldsymbol{L}}$ | 2.25 mS/cm^2^ |
| --- | --- |
| $\boldsymbol{E}_{\boldsymbol{L}}$ | -60 mV |
| ${\bar{\boldsymbol{g}}}_{\boldsymbol{Na}}$ | 37.5 mS/cm^2^ |
| $\boldsymbol{E}_{\boldsymbol{Na}}$ | 55 mV |
| ${\bar{\boldsymbol{g}}}_{\boldsymbol{K}}$ | 45 mS/cm^2^ |
| $\boldsymbol{E}_{\boldsymbol{K}}$ | -80 mV |
| ${\bar{\boldsymbol{g}}}_{\boldsymbol{Ca}}$ | 0.15 mS/cm^2^ |
| $\boldsymbol{E}_{\boldsymbol{Ca}}$ | 140 mV |
| ${\bar{\boldsymbol{g}}}_{\boldsymbol{T}}$ | 0.5 mS/cm^2^ |
| ${\bar{\boldsymbol{g}}}_{\boldsymbol{AHP}}$ | 9 mS/cm^2^ |
| $\boldsymbol{I}_{\boldsymbol{app}}$ | -15 uA |
| $\boldsymbol{I}_{\boldsymbol{z}}$ | ~$N\left( 0,0.1 \right) uA$ |

**STN cells**

| ${\bar{\boldsymbol{g}}}_{\boldsymbol{L}}$ | 0.05 mS/cm^2^ |
| --- | --- |
| $\boldsymbol{E}_{\boldsymbol{L}}$ | -70 mV |
| ${\bar{\boldsymbol{g}}}_{\boldsymbol{Na}}$ | 3 mS/cm^2^ |
| $\boldsymbol{E}_{\boldsymbol{Na}}$ | 50 mV |
| ${\bar{\boldsymbol{g}}}_{\boldsymbol{K}}$ | 5 mS/cm^2^ |
| $\boldsymbol{E}_{\boldsymbol{K}}$ | -90 mV |
| ${\bar{\boldsymbol{g}}}_{\boldsymbol{T}}$ | 5 mS/cm^2^ |
| $\boldsymbol{E}_{\boldsymbol{T}}$ | 0 mV |
| ${\bar{\boldsymbol{g}}}_{\boldsymbol{e}}$ | 0.05 mS/cm^2^ |
| $\boldsymbol{E}_{\boldsymbol{e}}$ | 0 mV |
| $\boldsymbol{I}_{\boldsymbol{z}}$ | ~$N\left( 0,0.1 \right) uA$ |

**TC cells**

**Table 2. Model cell parameters.** Maximum conductance and reversal potentials for the leak current and ionic currents are given above, along with parameters for other extraneous inputs to the cell models.

**Synaptic current parameters**

All synaptic currents are functions of the gating variable and reversal potential of the synapses, as well as a sum of presynaptic variables which change as a function of time. The presynaptic variables, *s_i_*, for each presynaptic cell are updated in every time step and control how much each cell contributes to the change in voltage potential in the postsynaptic cell. If the presynaptic cell type is *x* and the postsynaptic cell type is *y*, then the synaptic current into *y* from the all cells of type *x* is

$$i_{x\to y}= g_{x\to y}(v-E_{x\to y})\sum_{i\in x} s_{i}.$$

The functions for how *s_i_* are as follows:

$$h_{\infty}=\left( 1+ e^{-\frac{v- \theta_{g}- \theta_{g}^{H}}{\sigma_{g}^{H}}} \right)^{-1},$$

$$\frac{{\partial s}_{i}}{\partial t}=\alpha h_{\infty}\left( 1-s_{i} \right)- \beta s_{i}.$$

Below is a table for the synaptic variables of each type of synapse.

| Parameter | Value |
| --- | --- |
| $\boldsymbol{\theta}_{\boldsymbol{g}}$ | 20 |
| $\boldsymbol{\theta}_{\boldsymbol{g}}^{\boldsymbol{H}}$ | -39 |
| $\boldsymbol{\sigma}_{\boldsymbol{g}}^{\boldsymbol{H}}$ | 8 |
| $\boldsymbol{\alpha}$ | 2 ms^-1^ |
| $\boldsymbol{\beta}$ | 0.04 ms^-1^ |
| $\boldsymbol{g}_{\boldsymbol{GPe\to STN}}$ | 0.45 mS/cm^2^ |
| $\boldsymbol{E}_{\boldsymbol{GPe\to STN}}$ | -100 mV |

**GPe →STN**

| $\boldsymbol{\theta}_{\boldsymbol{g}}$ | 20 |
| --- | --- |
| $\boldsymbol{\theta}_{\boldsymbol{g}}^{\boldsymbol{H}}$ | -57 |
| $\boldsymbol{\sigma}_{\boldsymbol{g}}^{\boldsymbol{H}}$ | 2 |
| $\boldsymbol{\alpha}$ | 1 ms^-1^ |
| $\boldsymbol{\beta}$ | 0.04 ms^-1^ |
| $\boldsymbol{g}_{\boldsymbol{GPe\to GPe}}$ | 1 mS/cm^2^ |
| $\boldsymbol{E}_{\boldsymbol{GPe\to GPe}}$ | -70 mV |

**GPe →GPe**

| $\boldsymbol{\theta}_{\boldsymbol{g}}$ | 20 |
| --- | --- |
| $\boldsymbol{\theta}_{\boldsymbol{g}}^{\boldsymbol{H}}$ | -57 |
| $\boldsymbol{\sigma}_{\boldsymbol{g}}^{\boldsymbol{H}}$ | 2 |
| $\boldsymbol{\alpha}$ | 1 ms^-1^ |
| $\boldsymbol{\beta}$ | 0.04 ms^-1^ |
| $\boldsymbol{g}_{\boldsymbol{GPe\to GPi}}$ | 1 mS/cm^2^ |
| $\boldsymbol{E}_{\boldsymbol{GPe\to GPi}}$ | -70 mV |

**GPe →GPi**

| $\boldsymbol{\theta}_{\boldsymbol{g}}$ | 30 |
| --- | --- |
| $\boldsymbol{\theta}_{\boldsymbol{g}}^{\boldsymbol{H}}$ | -57 |
| $\boldsymbol{\sigma}_{\boldsymbol{g}}^{\boldsymbol{H}}$ | 2 |
| $\boldsymbol{\alpha}$ | 5 ms^-1^ |
| $\boldsymbol{\beta}$ | 1 ms^-1^ |
| $\boldsymbol{g}_{\boldsymbol{STN\to GPe}}$ | 0.015 mS/cm^2^ |
| $\boldsymbol{E}_{\boldsymbol{STN\to GPe}}$ | 0 mV |

**STN →GPe**

| $\boldsymbol{\theta}_{\boldsymbol{g}}$ | 30 |
| --- | --- |
| $\boldsymbol{\theta}_{\boldsymbol{g}}^{\boldsymbol{H}}$ | -57 |
| $\boldsymbol{\sigma}_{\boldsymbol{g}}^{\boldsymbol{H}}$ | 2 |
| $\boldsymbol{\alpha}$ | 1 ms^-1^ |
| $\boldsymbol{\beta}$ | 0.05 ms^-1^ |
| $\boldsymbol{g}_{\boldsymbol{STN\to GPi}}$ | 0.5 mS/cm^2^ |
| $\boldsymbol{E}_{\boldsymbol{STN\to GPi}}$ | 0 mV |

**STN →GPi**

| $\boldsymbol{\theta}_{\boldsymbol{g}}$ | 20 |
| --- | --- |
| $\boldsymbol{\theta}_{\boldsymbol{g}}^{\boldsymbol{H}}$ | -39 |
| $\boldsymbol{\sigma}_{\boldsymbol{g}}^{\boldsymbol{H}}$ | 2 |
| $\boldsymbol{\alpha}$ | 2 ms^-1^ |
| $\boldsymbol{\beta}$ | 0.08 ms^-1^ |
| $\boldsymbol{g}_{\boldsymbol{GPi\to TC}}$ | 1.5 mS/cm^2^ |
| $\boldsymbol{E}_{\boldsymbol{GPi\to TC}}$ | -85 mV |

**GPi→TC**

| $\boldsymbol{\theta}_{\boldsymbol{g}}$ | 30 |
| --- | --- |
| $\boldsymbol{\theta}_{\boldsymbol{g}}^{\boldsymbol{H}}$ | -57 |
| $\boldsymbol{\sigma}_{\boldsymbol{g}}^{\boldsymbol{H}}$ | 2 |
| $\boldsymbol{\alpha}$ | 5 ms^-1^ |
| $\boldsymbol{\beta}$ | 1 ms^-1^ |
| $\boldsymbol{g}_{\boldsymbol{PY\to IN}}$ | 0.0124 mS/cm^2^ |
| $\boldsymbol{E}_{\boldsymbol{PY\to IN}}$ | 0 mV |

**PY→IN**

| $\boldsymbol{\theta}_{\boldsymbol{g}}$ | 20 |
| --- | --- |
| $\boldsymbol{\theta}_{\boldsymbol{g}}^{\boldsymbol{H}}$ | -39 |
| $\boldsymbol{\sigma}_{\boldsymbol{g}}^{\boldsymbol{H}}$ | 2 |
| $\boldsymbol{\alpha}$ | 1 ms^-1^ |
| $\boldsymbol{\beta}$ | 0.8 ms^-1^ |
| $\boldsymbol{g}_{\boldsymbol{IN\to PY}}$ | 0.1735 mS/cm^2^ |
| $\boldsymbol{E}_{\boldsymbol{IN\to PY}}$ | -75 mV |

**IN→PY**

**Table 3. Synaptic current parameters.** Parameters for computing relevant presynaptic and synaptic variables are included above for all modeled excitatory and inhibitory synapses.
